# PPARγ Ameliorates *Mycobacterium tuberculosis* H37Ra-Induced Foamy Macrophage Formation via the ABCG1-Dependent Cholesterol Efflux Pathway in THP-1 Macrophages

**DOI:** 10.3389/fmicb.2022.829870

**Published:** 2022-03-31

**Authors:** Yutao Ye, Jun Liu, Yang Guo, Yujie Gao, Jiayue Rao, Rigu Su, Lu Zhang, Zikun Huang, Qing Luo, Junming Li

**Affiliations:** Jiangxi Province Key Laboratory of Laboratory Medicine, Department of Clinical Laboratory, The First Affiliated Hospital of Nanchang University, Nanchang, China

**Keywords:** tuberculosis, PPARγ, foamy macrophage, ABCG1, cholesterol efflux

## Abstract

Foamy macrophages are present during the course of *Mycobacterium tuberculosis* (*Mtb*) infection and seems to be nutrient-rich reservoir and secure reservoir for the bacilli, which leads to bacterial persistence and infection transmission. Peroxisome proliferator activated receptor γ (PPARγ) is a key transcription factor for cholesterol metabolism in macrophages and its role in regulating atherosclerosis related foamy macrophages (FMs) formation has been well-studied. However, knowledge about the mechanism of PPARγ regulating *Mtb* infection induced FM formation remains very limited. In this study, we investigate the functional role of PPARγ in *Mtb* H37Ra infection-induced foamy macrophages formation. H37Ra infection induced a time-dependent decreased expression of PPARγ that paralleled the augmented lipid body formation in THP1-derived macrophages. PPARγ antagonist GW9662 significantly potentiate H37Ra induced lipid body formation and inhibit ABCG1 expression, overexpression of ABCG1 by transduced macrophages with lentivirus significantly reversed the promotion effect of GW9662 on FM formation. Moreover, Treatment with a TLR2 neutralizing antibody ameliorated the activation of ABCG1 by *Mtb* H37Ra without significantly effecting the suppression of PPARγ, suggesting a greater role for TLR2 to regulate ABCG1 compared to PPARγ. Overall, this study showed that PPARγ is involved in ameliorating FM formation by regulating ABCG1 expression, these observations expose a novel role of PPARγ in the *Mtb* infection induced FM formation.

## Introduction

Tuberculosis (TB) is a chronic infectious disease caused by *Mycobacterium tuberculosis* (*Mtb*) infection. Once *Mtb* bacilli are inhaled, macrophages phagocytose them and become the primary niche for *Mtb* (Weiss and Schaible, 2015; Zhang et al., 2021). *Mtb*-infected foamy macrophages (FMs) represent the hallmark of TB lesions. FM refers to macrophages that phagocytose excess lipids and have bubble-like lipid bodies in their cytoplasm (Shim et al., 2020). Numerous studies have shown that the morphology and function of macrophages are altered after their conversion to FM; morphologically, intracellular lipids accumulate and take on a foamy shape; functionally, FM shows a reduced capacity for phagocytosis and antimicrobial activity (Agarwal et al., 2020); they also provide a nutrient source for *Mtb*, thus facilitating the long-term survival of *Mtb* in bodies (Peyron et al., 2008; Singh et al., 2017). In fact, FM is mainly located in the granuloma environment. The death of FMs and the release of their lipid droplets lead to the formation of a caseum and the spread of infection, which accelerate the transformation of latent infection into active tuberculosis (Russell et al., 2009). As FM plays a central role in tuberculosis development and infection dissemination, determining the mechanism of FM biogenesis is of great importance. However, the events involved in tuberculous foam cell formation remain unclear.

*Mycobacterium tuberculosis* and bacterial factors manipulate the lipid metabolism of macrophages in multiple ways, thereby promoting FM formation. For example, ESAT-6 stimulates the G protein-coupled receptor GPR109A, leading to enhanced glucose metabolism in macrophages and increased lipid synthesis (Singh et al., 2015). *Mtb* also induces the activation of both mTORC1 signaling and the caspase cascade to promote TAG synthesis (Guerrini et al., 2018). In addition, *Mtb* modulates nuclear transcription receptors of host cells involved in nuclear transcription. For instance, *Mtb* activates nuclear receptors such as PPARγ, TR4, and LXR and membrane receptors such as CD36, SR-A1, and ABCG1 to regulate the process of FM formation (Dodd et al., 2016; Lovewell et al., 2016; Ahsan et al., 2018), and these molecules are the main regulators of lipid metabolism in macrophages.

Peroxisome proliferator-activated receptor gamma (PPARγ) is a member of the lipid-activated nuclear receptor superfamily and plays a central role in macrophage lipid metabolites (Leopold et al., 2019). For example, in atherosclerosis, PPARγ promotes the expression of CD36, causing enhanced uptake of lipids by macrophages (Kotla et al., 2017); it also accelerates cholesterol efflux by upregulating the expression of ABCA1 and ABCG1 (Wang et al., 2018). In addition, it has been reported that enlarged atherosclerotic plaques in PPARγ knockout mice are associated with reduced ABCG1 expression (Chawla et al., 2001), indicating that the role of PPARγ in lipid efflux exceeds that in lipid uptake. In contrast, research on the role of PPARγ in tuberculosis has focused on its effect on lipid uptake. However, the relative lipid composition of FM differs in disease contexts (Guerrini et al., 2018; Guerrini and Gennaro, 2019), leading to a disease-specific mechanism of FM formation and a disease-specific role for PPARγ. Based on the above, the specific mechanistic link between PPARγ and the tuberculosis-related foamy cell phenotype is still being elucidated.

In this study, an *in vitro Mtb*-infected macrophage mode was established as described previously (Agarwal et al., 2020) to investigate the effect of *Mtb* infection on PPARγ expression and FM formation. The impacts of PPARγ activity on the formation of FM and the related mechanisms were also investigated. We found that *Mtb* H37Ra infection reduced PPARγ expression and that PPARγ is involved in ameliorating lipid accumulation through the regulation of ABCG1 expression.

## Materials and Methods

### Bacterial Strains and Culture Conditions

The *Mycobacterium tuberculosis* H37Ra strain was purchased from ATCC (25177), and the bacteria were grown at 37 °C in Middlebrook 7H9 medium (BD company, United States), supplemented with 10% albumin-dextrose-catalase (BD company, United States), 0.5% glycerol, and 0.05% Tween-80 (Sigma-Aldrich). The bacterial aggregates were disassociated and adjusted to an OD600 of 0.5 (approximately 10^7^ individual bacteria/mL).

### THP-1 Cells and *in vitro* Infection

Human promonocytic THP-1 cells (ATCC, TIB-202, Manassas, VA, United States) were cultured in RPMI-1640 medium alone with 10% FBS (Sigma–Aldrich), 100 U/mL penicillin and 100 μg/mL streptomycin and incubated at 37°C in a humidified atmosphere with 5% CO_2_. To induce THP-1 cells into macrophages, cells were adhered to cover slides within culture plates (24 wells) and exposed to phorbol 12-myristate 13-acetate (PMA, 50 nM, Sigma-Aldrich) for 24 h.

Macrophages were infected with H37Ra (MOI = 10:1) for 4 h, followed by three PBS washes to remove extracellular bacteria. When indicated, macrophages were pretreated with PPARγ agonist BRL49653 (5 μM, Cayman, MI, United States) or PPARγ antagonist GW9662 (1 μM, Sigma-Aldrich) for 30 min, or neutralizing antibody against TLR2 (h-TLR2, 5 μg/ml, Invivogen, San Diego, CA, United States) for 1 h before H37Ra infection.

### Oil Red O Staining

Cells were washed with PBS for three times and fixed in 4% paraformaldehyde for 30 min. Thereafter, cells were rinsed with 60% isopropanol and stained with fresh filtered Oil Red O (ORO, Sigma-Aldrich) solution for 15 min. Next, cells were washed with isopropanol (60%) followed by counterstaining with hematoxylin for 2 min. Cells were visualized under a microscope, and images were obtained. For the determination of intracellular lipid droplets, images of ORO-stained cells were quantified with ImageJ software. For quantification, three images from each treatment group were randomly selected, and the percentage of lipid droplets to total area was analyzed, then normalized to the total number of THP-1.

### Quantitative RT–PCR

Total RNA from the macrophages was extracted with TRIzol reagent (Ambion, Thermo Fisher Scientific, Waltham, MA, United States). The mRNA was reverse transcribed to cDNA using a PrimeScript RT Reagents kit (RR047A, Takara, Japan). qPCR was performed using SYBR Premix Ex Taq II (RR420A, Takara, Japan) in ABI7500, and the cycles were performed as follows: 30 s at 95°C, 40 cycles of 5 s at 95°C, and 30 s at 60°C. GAPDH was used as the housekeeping gene. Gene expression was calculated as 2^–ΔΔCT^. The real-time RT–PCR oligonucleotide primers are shown in Table 1.

**TABLE 1:** The real-time RT-PCR oligonucleotide primers.

Gene	Primer	Sequence (5′–3′)	PCR product (bp)
GAPDH	Forward	5′-GGGAGCCAAAAGGGTCATCA-3′	
(NM_001357943.2)	Reverse	5′-TGATGGCATGGACTGTGGTC-3′	184
PPARγ	Forward	5′-ACTTTGGGATCAGCTCCGTG-3′	
(NM_005037.7)	Reverse	5′-GGAGATGCAGGCTCCACTTT-3′	194
CD36	Forward	5′-TTGGGAAAGTCACTGCGACA-3′	
NM_001371075.1	Reverse	5′-TCAACTGGAGAGGCAAAGGC-3′	163
LDLR	Forward	5′-AGAAGAAGCCCAGTAGCGTG-3′	
(NM_000527.5)	Reverse	5′-CTGTCTCGAGGGGTAGCTGT-3′	192
SR-A1	Forward	5′-AGCCCACAAGTTTCCCAGTC-3′	
(NM_001035235.4)	Reverse	5′-GGCTTGAAAGCTCTTGCACC-3′	196
ABCG1	Forward	5′-CTGTCTGATGGCCGCTTTCT-3′	
(NM_016818.3)	Reverse	5′-AATGTTCACAGCTGCCCTCC-3′	216
ABCA1	Forward	5′-ATTCCTCAAGGTGGCCGAAG-3′	
(NM_005502.4)	Reverse	5′-CCTTTGCCATCCATCCCACT-3′	184
SR-B1	Forward	5′-ATGCACTATGCCCAGTACGTC-3′	
(NM_005505.5)	Reverse	5′-TTTGCTTCCTGCAGCACAGAG-3′	182

### Western-Blot

Macrophages were lysed in RIPA lysis buffer (Solarbio, Beijing, China). Protein concentrations were measured using a BCA protein assay kit (Solarbio, Beijing, China). Equal amounts of protein (30 μg) were loaded onto 10% gels. Separated proteins were transferred to PVDF membranes membrane (Millipore, Billerica, MA, United States); subsequently, the membranes were blocked with 5% non-fat milk for 2 h and probed with antibodies against PPARγ (1:1,000 dilution, C26H12, Cell Signaling Technology, Danvers, MA, United States), ABCG1 (1:1,000 dilution, ab52617, ABcam, Cambridge, MA, United States) or TLR2(1:1,000 dilution, 66645-1, Sanying, Wuhan, China) overnight at 4°C. Thereafter, the membranes were washed with TBS-Tween buffer and further incubated with an HRP-conjugated goat anti-rabbit IgG Ab (1:5,000, ab205718, ABcam, Cambridge, MA, United States) or HRP-conjugated goat anti-mouse IgG Ab (1:5,000, ab150113, ABcam, Cambridge, MA, United States) at room temperature for 2 h. Protein bands were visualized by an enhanced chemiluminescence detection kit (Thermo Fisher Scientific, Waltham, MA, United States) and quantified with ImageJ software.

### Construction of the Lentiviral Vector

The ABCG1 gene was amplified by PCR and inserted into the PLVX-IRES-ZS-GREEN1 vector. The newly generated plasmid was LV-ABCG1, and PLVX-IRES-ZS-GREEN was LV-ctl. The cytomegalovirus (CMV) promoter was used to drive gene expression. The packaging plasmids psPAX2 and enveloped protein particles PMD2.G were transfected into 293T cells for lentiviral production. The lentiviruses were collected on Day 3 after the transfection and concentrated by ultracentrifugation. THP-1 cells were exposed to lentiviruses at a MOI of 50 and 8 μg/ml polybrene (Sigma-Aldrich). Gene transduction efficiency was analyzed by flow cytometry. RT–PCR and western blotting were performed to detect ABCG1 expression in PLVX-ABCG1-transduced THP-1 cells at 3 days after transduction.

### Statistical Analysis

Data were derived from 3 or more independent experiments and are shown as the means ± standard deviation (SD). The results were analyzed using GraphPad Prism 8.0 software. For non-parametric unpaired data, comparisons were made using the Mann–Whitney test. For parametric unpaired data, comparisons were determined by Student’s *t*-test. Statistical significance was taken as *p* < 0.05. *P*-values were assigned as *P* > 0.05; *P ≤ 0.05; **P ≤ 0.01; ***P ≤ 0.001; and ****P ≤ 0.0001.

## Results

### *Mycobacterium tuberculosis* Infection Induces Foamy Macrophage Formation

**FIGURE 1:**
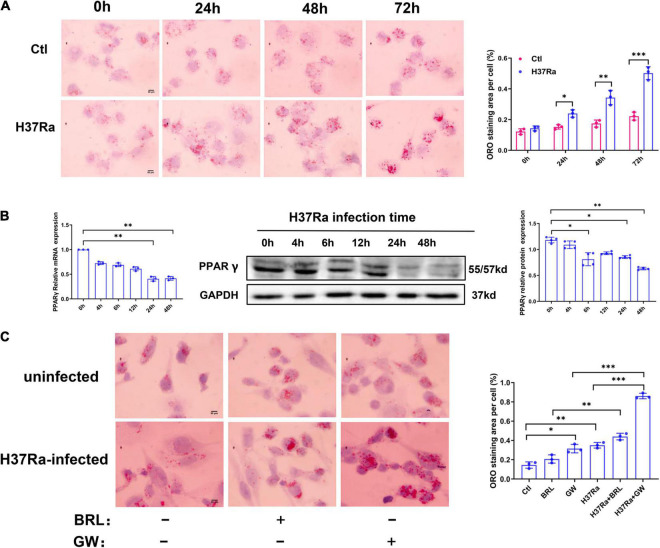
Peroxisome proliferator activated receptor γ (PPARγ) is involved in *Mycobacterium tuberculosis* (*Mtb*)-induced foamy macrophage (FM) formation. THP-1 cells were infected with (H37Ra group) or without (Ctl group) H37Ra (MOI = 10:1) and were cultivated for the indicated time points. Representative images of ORO-stained macrophages are presented [**(A)**, left panel, 1,000×]. Intracellular lipid drops were quantified with ImageJ software [**(A)**, right panel]. THP-1 cells were infected with H37Ra (MOI = 10:1) or 4 h, washed three times to remove extracellular Mtb and cultivated for different time points, then harvested for the detection of PPARγ expression by RT–qPCR and western blot **(B)**. THP-1 cells were pretreated with a PPARγ agonist BRL49653 (5 μM) or antagonist GW966 2 (1 μM) for 30 min, followed by infection with H37Ra for 72 h and staining with ORO [**(C)**, 1,000×]. Scale bar = 50 μm. Data are expressed as the means ± SD of at least three independent experiments, *P < 0.05, **P < 0.01, ***P < 0.001.

THP-1-derived macrophages were infected with *Mycobacterium tuberculosis* H37Ra, and the accumulation of intracellular lipid drops was detected by ORO staining till 72 h after infection. The results showed that when compared with uninfected cells, *Mtb* H37Ra infection significantly upregulated lipid accumulation in macrophages, with the most prominent effect observed at 72 h after infection (Figure 1A).

### Peroxisome Proliferator Activated Receptor γ Is Involved in *Mycobacterium tuberculosis*-Induced Foamy Macrophage Formation

We next examined the effect of H37Ra infection on PPARγ expression in THP-1 macrophage-derived foam cells. Macrophages were infected with *Mtb* H37Ra for different time points and then detected for PPARγ mRNA and protein expression. As shown in Figure 1B, compared with the uninfected control group (0 h), the expression of PPARγ gradually decreased with prolonged infection time, which demonstrated that H37Ra infection induced a time-dependent decrease in PPAR expression in macrophages.

To investigate the role of PPARγ in lipid accumulation within *Mtb* H37Ra-infected macrophages, we pretreated macrophages with a PPARγ agonist BRL49653 or antagonist GW9662 followed by H37Ra infection. As shown in Figure 1C, GW9662 significantly exacerbated the accumulation of lipids in macrophages. Pretreatment with BRL49653 had a minimal effect on lipid accumulation in macrophages.

### The Role of Peroxisome Proliferator Activated Receptor γ in Regulating Foamy Macrophage Formation Is Related to the Regulation of ABCG1 Expression

**FIGURE 2:**
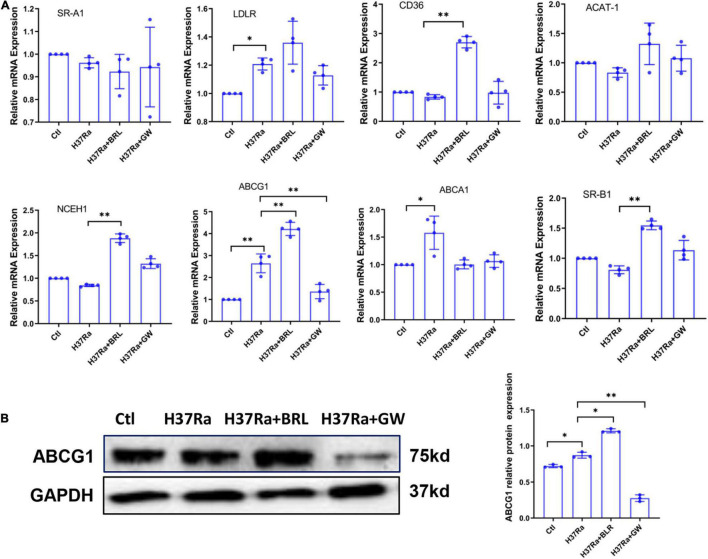
Effect of peroxisome proliferator activated receptor γ (PPARγ) activity on the expression of lipid metabolism-related molecules. THP-1 cells were pretreated with BRL49653 (5 μM) or GW9662 (1 μM) for 30 min followed by infection with H37Ra (MOI = 10:1) for 24 h. The mRNA levels of SR-A1, LDLR, CD36, ACAT-1, NCEH1, ABCA1, ABCG1, and SR-B1 were detected by RT–PCR **(A)**. The protein expression of ABCG1 was determined by western blot **(B)** and quantified by grayscale scanning with ImageJ software. Data are expressed as the means ± SD of at least three independent experiments, *P < 0.05, **P < 0.01.

To explore the molecular mechanism of PPARγ in regulating FM formation, we detected the expression of molecules involved in the influx and efflux of lipids under conditions of PPARγ activity regulation. The results from the RT–PCR assay showed that H37Ra infection significantly enhanced the expression of LDLR, ABCG1, and ABCA1 compared to the non-infection control group; the PPARγ agonist BRL49653 significantly increased the expression of CD36, NCEH1, ABCG1 and SR-B1, and the PPARγ antagonist GW9662 downregulated the expression of ABCG1 (Figure 2A). Next, we verified the effect of PPARγ activity regulation on ABCG1 expression by western blot, and the results were consistent with those of RT–PCR (Figure 2B).

**FIGURE 3:**
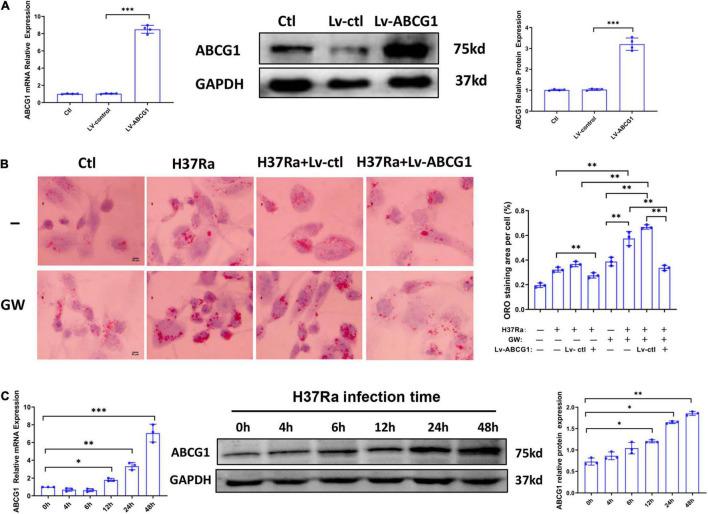
The role of peroxisome proliferator activated receptor γ (PPARγ) in regulating foamy macrophage (FM) formation is related to the regulation of ABCG1 expression. THP-1 cells were transduced with Lv-ctl or Lv-ABCG1 for 48 h, followed by the detection of ABCG1 expression by both RT–PCR and western blot **(A)**. THP-1 cells were transduced with Lv-ctl or Lv-ABCG1 for 72 h, treated with GW9662 for 30 min, infected with H37Ra (MOI = 10:1) for 72 h and then evaluated for intracellular lipid accumulation by ORO staining [**(B)**, left panel, 1,000×]. Scale bar = 50 μm. The intracellular lipid bodies were quantified with ImageJ software [**(B)**, right panel]. Effects of *Mycobacterium tuberculosis* (*Mtb*) infection on ABCG1 expression in THP-1 cells were detected. THP-1 cells were infected with H37Ra for the indicated times and then harvested for the detection of ABCG1 expression by RT–qPCR and western blot **(C)**. Data are expressed as the means ± SD of at least three independent experiments. *P < 0.05, **P < 0.01, ***P < 0.001.

Based on the above, we sought to determine whether GW9662 promoted FM formation by inhibiting ABCG1 expression. THP-1 cells were transduced with lentivirus overexpressing ABCG1 (Lv-ABCG1) or control lentivirus (Lv-ctl) (Figure 3A), pretreated with or without GW9662, infected with H37Ra (MOI = 10) and monitored for the accumulation of intracellular lipids. The results showed that when compared with H37Ra-infected group, Lv-ABCG1 significantly downregulated lipid accumulation in macrophages, while Lv-ctl had a minimal effect on lipid accumulation. GW9662 treatment significantly promoted lipid accumulation in H37Ra-infected group and Lv-ctl group, while Lv-ABCG1 reversed the promoting effect of GW9662 on lipid accumulation in H37Ra infection group and Lv-ctl group (Figure 3B). We also investigated the effect of *Mtb* H37Ra infection on ABCG1 expression in THP-1 cells. The results showed that *Mtb* H37Ra infection increased ABCG1 expression in a time-dependent manner (Figure 3C).

### Peroxisome Proliferator Activated Receptor γ Expression During H37Ra Infection Is Independent of TLR2 Activation

**FIGURE 4:**
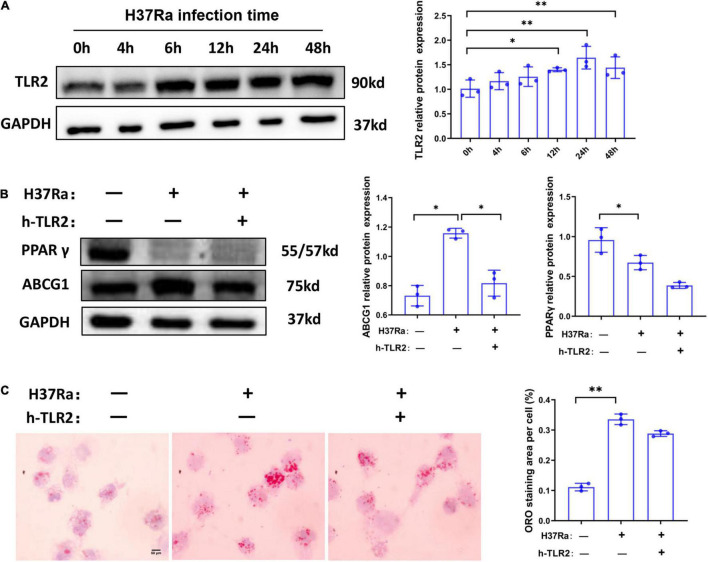
The expression change of peroxisome proliferator activated receptor γ (PPARγ) in macrophages in the course of *Mycobacterium tuberculosis* (*Mtb*) infection is TLR2-independent. THP-1 cells were infected with H37Ra for the indicated times and then harvested for the detection of TLR2 expression by western blot **(A)**. THP-1 cells were pretreated with h-TLR2 (5 μg/ml) for 1 h, followed by infection with H37Ra (MOI = 10:1), and the protein expression levels of PPARγ and ABCG1 were determined by western blot **(B)**, the intracellular lipid accumulation were evaluated by ORO staining [**(C)**, 1,000×]. Scale bar = 50 μm. Data are expressed as the means ± SD of at least three independent experiments, *P < 0.05, **P < 0.01.

TLR2 has been demonstrated to be a key regulator of macrophage immunity and FM formation, we therefore wanted to know whether TLR2 is involved in the regulation of PPARγ and ABCG1 expression in the course of *Mtb* H37Ra infection. As shown in Figure 4A, H37Ra infection elevated the expression of TLR2 in macrophages. Considering that H37Ra infection was found to suppress the expression of PPARγ, it seemed that TLR2 signaling could downregulate the expression of PPARγ. However, blocking the TLR2 signal with a neutralizing antibody (polyclonal anti-hTLR2 antibody, h-TLR2) against TLR2 before *H37Ra* infection did not promote the expression of PPARγ. In contrast, it inhibited the expression of PPARγ and ABCG1 (Figure 4B), which demonstrated that the decrease in PPARγ expression in the course of *Mtb* H37Ra infection is TLR2-independent. Besides, ORO staining showed that compared with H37Ra-infected group, h-TLR2 treatment had no significant effect on lipid accumulation in macrophages (Figure 4C).

## Discussion

Foamy macrophages are associated with chronic inflammation in metabolic, infectious, or autoimmune diseases, and their functional role and formation mechanisms have been most well-studied in atherosclerosis. Previous studies have shown that FM is present in tuberculous lesions of animal models as well as in granulomas of individuals with active tuberculosis (Peyron et al., 2008; Gago et al., 2018). Our present study confirmed that *Mtb* induces FM formation in a time-dependent manner in an *in vitro Mtb*-infected macrophage model. Since *Mtb*-infected foamy macrophages represent the major niche for *Mtb* survival and their presence is associated with the activation of tuberculosis, FM is likely to determine the prognosis and regression of tuberculosis, but to date, the mechanisms of TB-associated FM formation need to be further investigated.

PPARγ is one of the key receptors regulating lipid metabolism and is involved in regulating the formation of FM in different disease contexts. Previous studies have implicated PPARγ in the regulation of CD36 expression and the uptake of ox-LDL. In atherosclerosis, PPARγ is reported to bind with the promoter region of CD36, and the PPARγ agonist BLR49653 strongly induces CD36 expression (Rubic and Lorenz, 2006; Kotla et al., 2017). During tuberculosis, BLR49653 treatment had the same effect on CD36 expression and FM formation (Mahajan et al., 2012); in silicosis, PPARγ antagonist GW9662 treatment downregulated CD36 expression and inhibited FM formation (Hou et al., 2019). Subsequent studies reported the functional role of PPARγ in regulating lipid efflux. In alveolar proteinosis, PPARγ regulates the formation of FM by regulating the expression of ABCG1 (Malur et al., 2011). In an *in vitro* atherosclerosis cell model, allicin, leonurine, etc. have been shown to promote lipid efflux through the PPARγ-LXRα-ABCG1 axis (He et al., 2016; Lin et al., 2017). Moreover, a study by Ajay Chawla et al. found that deletion of the PPARγ gene in macrophages enlarged atherosclerotic plaques in LDLR-/- mice (Chawla et al., 2001), demonstrating that in atherosclerosis, the effect of PPARγ in promoting lipid efflux exceeded that in lipid uptake. What is the main function of PPARγ in the formation of tuberculosis-associated FM?

In this study, we confirmed that H37Ra infection decreased PPARγ expression in a time-dependent manner in THP-1 macrophage. Inconsistent with our results, a study by Sahil Mahajan et al. showed that PPARγ expression was altered in a fluctuating manner in THP-1 cells within 24 h after H37Ra infection (Mahajan et al., 2012). H37Rv infection or BCG infection led to increased PPARγ expression, and non-pathogenic *Mycobacterium smegmatis* failed to induce PPARγ expression (Almeida et al., 2009; Dasgupta and Rai, 2018). Based on the above, we conclude that the relative expression of PPARγ varies with bacterial strains for the following reasons: (i) *Mtb* of the same type or even different source strains of the same type have different effects on PPARγ activation due to different levels virulence, and (ii) PPARγ expression varies with MOI and infection time. These findings also imply that PPARγ is involved in regulating the immune response against TB.

On this basis, we asked whether PPARγ activation is involved in the regulation of FM formation. We observed that the PPARγ antagonist GW9662 potentiated lipid body accumulation and that the PPARγ agonist BRL49653 had a minimal effect on FM formation. To further investigate the molecular mechanism involved, we analyzed the effect of PPARγ activation on the expression of lipid metabolism-related molecules. The results showed that BRL49653 promoted the expression of CD36 as well as ABCG1 and ABCA1, indicating that BRL49653 can promote not only lipid intake but also lipid efflux. Moreover, although it may accelerate lipid metabolism in macrophages, it has no significant effect on FM formation. GW9662 treatment significantly inhibited ABCG1 expression. Based on the above, we hypothesize that the promotion effect of GW9662 on FM formation may be related to its inhibition of ABCG1 expression. To confirm the involvement of ABCG1 in H37Ra-induced lipid body formation, the cells were infected with Lv-ABCG1 before GW9662 treatment followed by H37Ra infection. We found that the promotion effect of GW9662 on lipid accumulation was reversed by Lv-ABCG1. These observations confirmed our deduction and indicated that PPARγ decreases lipid accumulation by inducing ABCG1 expression. However, our results showed that H37Ra infection led to increased expression of ABCG1 and decreased expression of PPARγ in THP-1-derived macrophages in a time-dependent manner, suggesting that PPARγ activation, although involved in mycobacteria-induced ABCG1 expression, is not essential to trigger ABCG1 activation. Other cofactors may be involved in this process. Some related studies have shown that the ABCG1 gene is itself a direct target of LXRα and LXRα is a selective target of PPARγ in the context of atherosclerosis (Chawla et al., 2001; Rasheed and Cummins, 2018). As a consequence of this regulatory loop, both PPARγ and LXR ligands are involved in regulating ABCG1 expression. Therefore, in this study, whether LXRα is the main molecule targeting ABCG1 remains to be further investigated.

Our study reports that *Mtb* H37Ra infection results in a decrease in PPARγ expression accompanied by increased FM formation. GW9662 was an effective inducer of FM formation, decreased expression or weakened activity of PPARγ promotes the formation of FM in our *in vitro* cell model. The mechanism involved is related to the PPARγ/ABCG1 axis. In contrast, D’Avila et al. reported that BRL49653 significantly promotes lipid accumulation in mouse peritoneal macrophages after BCG infection (D’Avila et al., 2006). However, a study in a mouse atherosclerosis model revealed that PPARγ knockout mice exhibited enlarged atherosclerotic plaques, which is related to the suppression of ABCG1 expression (Chawla et al., 2001). Based on these findings, we speculate a possible lipid metabolism process in macrophages: initially, *Mtb* infection results in the induction of PPARγ and its target genes, including CD36, facilitating the uptake of triglycerides and ox-LDL and resulting in increased lipid accumulation. These excessive lipids then stimulate cellular cholesterol efflux by activating LXRs or other molecules to upregulate ABCG1 gene expression. Thus, PPARγ is proposed to couple a pathway of oxLDL uptake to efflux, thereby accelerating lipid flow from macrophages.

In addition, our findings demonstrate that TLR2 activation, although involved in inducing PPARγ expression, is not the central molecule in targeting PPARγ. The TLR family has been implicated in mycobacterial recognition and signaling pathways. In particular, TLR2 appears to be critical for sensing mycobacteria and is recognized as a principal inducer of signals in mycobacterial infection (Almeida et al., 2009; Leopold et al., 2019). Our results showed that TLR2 neutralizing antibody h-TLR2 result to decreased ABCG1 expression, but had no significant effect on PPARγ expression and lipid accumulation in macrophages. And h-TLR2 ameliorated the activation of ABCG1 without significantly effecting the suppression of PPARγ, suggesting a greater role for TLR2 to regulate ABCG1 compared to PPARγ, which imply a PPARγ-independent or compensatory pathway to ABCG1 that are mediated by TLR signaling. For instance, Ahsan et al. (2018) reported that IL-36/LXR axis is involved in modulating cholesterol metabolism during Mtb infection, LXR ligands induced cholesterol efflux whereas cholesterol efflux impaired in the presence of LXR inhibitors. Besides, our results show that *Mtb H37Ra* infection activates TLR2 and simultaneously inhibits PPARγ expression in a time-dependent manner, indicating that other pattern recognition receptors (PRRs) may be involved in regulating PPARγ other than TLR2. In fact, different members of the TLR family, including TLR1, TLR4, and TLR6, as well as TLR9, were demonstrated to act as mycobacterial signaling transmitters (Saraav et al., 2014); accordingly, TLR2 may cooperate with these receptors to regulate the expression of PPARγ. Additional studies will be necessary to characterize the specific PPRs for PPARγ regulation involved in lipid body biogenesis.

Although the mechanism of PPARγ in regulating lipid efflux has been well-studied in atherosclerosis, in tuberculosis, it still needs to be further investigated. In the present study we provide evidence that PPARγ plays an important role in regulating lipid efflux in tuberculosis-related FM formation. However, this study still has some shortcomings. Since THP-1 cells are terminally differentiated cells, we modulated their activity by agonists and antagonists and failed to regulate their expression levels by siRNA or other treatments. Moreover, *in vitro* cell experiments cannot be compared with *in vivo* experiments; therefore, further validation by animal experiments is subsequently required. Future studies in animal models as well as in *M. tuberculosis* infection will be necessary for further characterizations.

**FIGURE 5:**
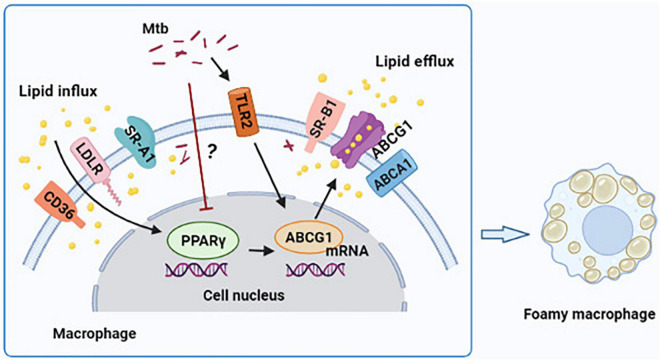
Graphical summary depicting that peroxisome proliferator activated receptor γ (PPARγ) regulates Mtb H37Ra infection-induced foamy macrophage formation via the ABCG1-dependent cholesterol efflux pathway. H37Ra infection reduces PPARγ expression in macrophage in a currently unknown way, which results in the decrease of ABCG1 expression and intracellular lipid efflux. TLR2 is involved in the regulation of PPARγ and ABCG1 expression, and it have a greater role to regulate ABCG1 compared to PPARγ, but it is not the central regulator of PPARγ expression.

In conclusion, our findings demonstrate that H37Ra infection reduces PPARγ expression and induces FM formation in a time-dependent manner in THP-1-derived foamy macrophages. Moreover, PPARγ ameliorates lipid accumulation through the regulation of ABCG1 expression; furthermore, TLR2 is not the central molecule in targeting PPARγ expression (Figure 5). These findings offer a new perspective on the participation of PPARγ in foamy macrophage formation.

## Data Availability Statement

The original contributions presented in the study are included in the article/supplementary material, further inquiries can be directed to the corresponding authors.

## Author Contributions

YY: data curation and writing–original draft preparation. JLu and LZ: methodology. YGu and RS: visualization. YGa and JR: investigation. QL: project administration. ZH: supervision. JLi: conceptualization and writing–review and editing. All authors contributed to the article and approved the submitted version.

## Conflict of Interest

The authors declare that the research was conducted in the absence of any commercial or financial relationships that could be construed as a potential conflict of interest.

## Publisher’s Note

All claims expressed in this article are solely those of the authors and do not necessarily represent those of their affiliated organizations, or those of the publisher, the editors and the reviewers. Any product that may be evaluated in this article, or claim that may be made by its manufacturer, is not guaranteed or endorsed by the publisher.
